# RETRACTION: Comparison of Preventive and Therapeutic Effects of Continuous Exercise on Acute Lung Injury Induced with Methotrexate

**DOI:** 10.1113/eph.13825

**Published:** 2025-03-20

**Retraction**: M.‐A. Rajizadeh, M. H. Hosseini, M. Bahrami, N. S. Hosseini, F. Rostamabadi, F. Bagheri, K. Khoramipour, H. Najafipour, and M.‐A. Bejeshk, “Comparison of Preventive and Therapeutic Effects of Continuous Exercise on Acute Lung Injury Induced with Methotrexate,” *Experimental Physiology *108, no. 9 (2023): 1215–1227, https://doi.org/10.1113/EP091162.

The above article, published online on 27 July 2023 in Wiley Online Library (wileyonlinelibrary.com), and its correction (https://doi.org/10.1113/EP091585), have been retracted by agreement between the journal Editor‐in‐Chief, Damian Bailey; The Physiological Society; and John Wiley & Sons Ltd. The retraction has been agreed following an investigation based on concerns raised by a third party (https://doi.org/10.1113/EP092385).

Concerns were raised that the control and continuous training (CT) tissue reported in Figure 7 incorrectly show adipose tissue instead of lung tissue, and the authors were unable to locate the original immunohistochemistry slides upon request. There are also concerns that the histology of the acute lung injury (ALI) group presented in Figure 6 does not resemble the alveoli with thickened walls presented in Figure 7, and that the magnification of these images differs. Finally, the dosages of ketamine and xylazine were not reported accurately in the methods section. As a result, the data and conclusions are considered unreliable, and the article therefore must be retracted.

